# Optimal antiplatelet therapy after revascularization of left subclavian artery during TEVAR

**DOI:** 10.1186/s13019-024-02932-3

**Published:** 2024-06-28

**Authors:** Mengxiao Shi, Hong Fang, Ying Wu, Han Li, Chong Sheng, Shuchun Li, Qing Zhou

**Affiliations:** 1 Department of Cardio-Thoracic Surgery, Nanjing Drum Tower Hospital Clinical College of Nanjing University of Chinese Medicine, Number 321 Zhong-shan Road, Nanjing, Jiangsu, 210008 China; 2 Department of Cardio-Thoracic Surgery, Affiliated Drum Tower Hospital, Medical School of Nanjing University, Nanjing, China; 3 Department of Cardio-Thoracic Surgery, Nanjing Drum Tower Hospital, Nanjing, China

**Keywords:** TEVAR, Left subclavian artery revascularization, Antiplatelet, Aspirin, Clopidogrel, Aortic dissection

## Abstract

**Background:**

Thoracic endovascular aortic repair (TEVAR) is a minimally invasive technique used to treat type B aortic dissections. Left subclavian artery (LSA) reconstruction is required when treating patients with involvement of LSA. The best antiplatelet therapy after LSA reconstruction is presently uncertain.

**Methods:**

This study retrospectively analyzed 245 type B aortic dissection patients who underwent left subclavian artery revascularization during TEVAR. Out of 245 patients, 159 (64.9%) were in the single antiplatelet therapy (SAPT) group, receiving only aspirin, and 86 (35.1%) were in the dual antiplatelet therapy (DAPT) group, receiving aspirin combined with clopidogrel. During the 6-month follow-up, primary endpoints included hemorrhagic events (general bleeding and hemorrhagic strokes), while secondary endpoints comprised ischemic events (left upper limb ischemia, ischemic stroke, and thrombotic events), as well as death and leakage events. Both univariate and multivariate Cox regression analyses were performed on hemorrhagic and ischemic events, with the Kaplan-Meier method used to generate the survival curve.

**Results:**

During the six-month follow-up, the incidence of hemorrhagic events in the DAPT group was higher (8.2% vs. 30.2%, *P* < 0.001). No significant differences were observed in ischemic events, death, or leakage events among the different antiplatelet treatment schemes. Multivariate Cox regression analysis showed that DAPT (HR: 2.22, 95% CI: 1.07–4.60, *P* = 0.032) and previous chronic conditions (HR:3.88, 95% CI: 1.24–12.14, *P* = 0.020) significantly affected the occurrence of hemorrhagic events. Chronic conditions in this study encompassed depression, vitiligo, and cholecystolithiasis. Carotid subclavian bypass (CSB) group (HR:0.29, 95% CI: 0.12–0.68, *P* = 0.004) and single-branched stent graft (SBSG) group (HR:0.26, 95% CI: 0.13–0.50, *P* < 0.001) had a lower rate of ischemic events than fenestration TEVAR (F-TEVAR). Survival analysis over 6 months revealed a lower risk of bleeding associated with SAPT during hemorrhagic events (*P* = 0.043).

**Conclusions:**

In type B aortic dissection patients undergoing LSA blood flow reconstruction after synchronous TEVAR, the bleeding risk significantly decreases with the SAPT regimen, and there is no apparent ischemic compensation within 6 months. Patients with previous chronic conditions have a higher risk of bleeding. The CSB group and SBSG group have less ischemic risk compared to F-TEVAR group.

## Introduction

Currently, TEVAR is widely acknowledged and has become the primary treatment for certain patients with thoracic descending aortic diseases [1, 2]. The European Society of Vascular Surgery and other guidelines recommend TEVAR as the primary treatment for descending thoracic aortic aneurysms (DTAA) [3–5]. Covering the left subclavian artery is advisable for patients lacking adequate proximal anchorage area. However, it increases the risks of stroke, spinal cord ischemia, and left upper limb ischemia [6]. In 2009, the Society of Vascular Surgery recommended routine revascularization when covering the left subclavian artery during TEVAR procedures [7]. Currently, clinical methods for LSA reconstruction, include carotid subclavian bypass (CSB), subclavian-carotid transposition (SCT) [8], fenestrated TEVAR(F-TEVAR) [9], single-branched stent graft(SBSG) [10], and other approaches. To mitigate adverse events of vascular embolism following left subclavian artery revascularization, practitioners often employ empirical antiplatelet therapy.

Aspirin is commonly employed in clinics to prevent cardiovascular and cerebrovascular events [11, 12], with its primary adverse reaction being bleeding [13, 14]. Dual antiplatelet therapy, combining aspirin and clopidogrel, is frequently utilized in secondary stroke prevention, reducing the risk of recurrent mild ischemic stroke and transient ischemic attack (TIA) [15, 16]. In their study assessing the safety and efficacy of antiplatelet therapy in patients with type B aortic dissection and coronary heart disease, He Ruixia found no significant difference in blood vessel patency between single antiplatelet therapy and dual antiplatelet therapies after endovascular abdominal aortic repair (EVAR). However, they observed an increased risk of bleeding with dual antiplatelet therapy, leading to their recommendation of long-term low-dose oral enteric aspirin. However it remains uncertain whether SAPT is superior to DAPT specifically for patients with type B aortic dissection [17]. Emma C Hansson and colleagues suggested that preoperative use of DAPT would elevate the risk of bleeding and blood transfusion, without impacting mortality. The study did not involve postoperative studies [18]. However, there are few reports on antiplatelet therapy after concomitant left subclavian artery revascularisation for TEVAR, the experience with SAPT does not directly extrapolate to this particular group of patients, and the optimal antiplatelet regimen remains unresolved.

Therefore, the objective of this study was to assess the efficacy and safety of both SAPT and DAPT regimens six months after simultaneous left subclavian artery revascularization for TEVAR. The aim is to provide clinicians with some insights to aid in selecting the most suitable antiplatelet regimen.

## Subjects and methods

### Study population

**Fig. 1:**
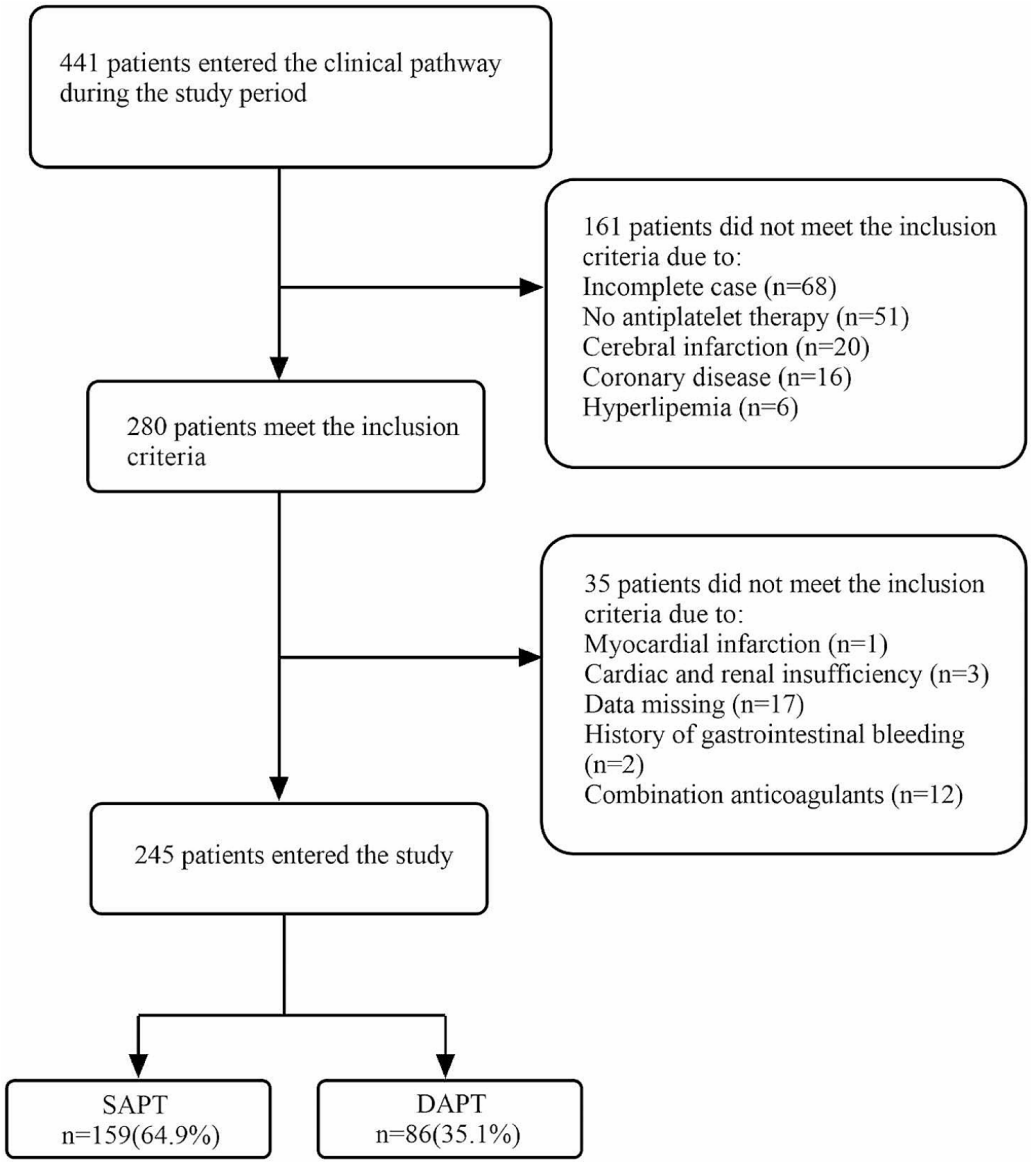
Consort diagram of patient screening and allocation

A retrospective analysis was conducted on patients who underwent TEVAR combined with left subclavian artery revascularization at Nanjing Drum Tower Hospital from January 2018 to May 2023. During the study of aspirin resistance, 441 patients were admitted to our center, and 245 type B aortic dissection patients were finally included in our study according to the standard of admission. Figure 1.

The inclusion criteria were as follows: (1) Patients undergoing TEVAR utilized F- TEVAR, SBSG and CSB for left subclavian artery reconstruction. They also received aspirin alone or in combination with clopidogrel after the operation. Exclusion criteria were: (1) patients diagnosed with stroke, coronary heart disease, myocardial infarction, hyperlipemia, hypertension combined with renal failure; (2) patients with a history of gastrointestinal bleeding or peptic ulcer disease; (3) patients with a history of major organ bleeding or severe bleeding disorders; (4) patients taking drugs that increase the risk of bleeding (such as non-steroidal anti-inflammatory drugs, corticosteroids, new oral anticoagulants and warfarin, etc.); (5) patients with severe heart, liver, and kidney dysfunction; (6) patients with coagulation disorders; (7) patients allergic to contrast agents; (8) any other condition that the researchers deemed unsuitable for inclusion in the study.

### Research methods

Patients with type B aortic dissection were categorized based on the postoperative use of antiplatelet drugs into the SAPT group (159 cases) and the DAPT group (86 cases). The SAPT group received (100 mg/day) aspirin as monotherapy, whereas the DAPT group received a combination of (100 mg/day) aspirin and (75 mg/day clopidogrel) for six months. Clinical data were collected from patients, who were then followed up after discharge through a combination of in-person interviews and telephone calls. Blood pressure, clinical symptoms, and aorta CTA were documented. Chronic conditions in this study encompassed depression, vitiligo, and cholecystolithiasis. General bleeding events include the upper digestive tract and eyes, etc., and the classification and definition standards are in accordance with the bleeding grading standards of the Bleeding Academic Research Consortium (BARC) [19]. Refer to Table 1 for more details. Categorization of patients’ surgical approaches based on LSA revascularization method: carotid-subclavian bypass (CSB), fenestrated TEVAR (F-TEVAR), and single-branched stent graft (SBSG).

**Table 1:** Bleeding grading standards of the Bleeding Academic Research Consortium [19]

Type	Clinical indications
1	Non-active bleeding, patients who do not need hospitalization; or patients who discontinue medication leading to bleeding
2	Any obvious active bleeding, meets one of the following conditions: requiring internal medical intervention, hospitalization, rapid assessment
3	Obvious bleeding with a decrease in hemoglobin of 3–5 g/dl; obvious bleeding requiring transfusion.
4	Not applicable
5	Fatal bleeding

The primary endpoint event is hemorrhagic events, classified according to the BARC classification. The secondary endpoints comprised death, leakage events and ischemic events (left upper limb ischemia, ischemic stroke, and thrombotic events). The thrombotic events encompassing the detection of stent filling defects, branching stents are not clear or in-stent thrombotic.

### Statistical method

SPSS 26.0 statistical software and R version 4.3.0 software were used for data analysis. The measurement data were expressed as mean ± standard deviation, and the count data were expressed as frequency and percentage. *T*-test was used for comparison and measurement data between the two groups, the *X*^2^ test was used for enumeration data, and the Fisher exact probability method was used for enumeration data with an expected frequency of less than 5. Univariate and multivariate Cox regression was used to analyze the influencing factors of bleeding events. Items with a *P* < 0.2 were included in the multifactorial analysis. *P* < 0.05 was considered statistically significant.

## Result

### Baseline information

The study included 245 type B aortic dissection patients, with 159 patients (64.9%) receiving aspirin monotherapy and 86 patients (35.1%) receiving aspirin in combination with clopidogrel dual antiplatelet therapy. The study comprised 86 cases of F-TEVAR, 124 cases of SBSG, and 35 cases of CSB. Significant differences were observed between the F-TEVAR group (*P* = 0.006) and the CSB group (*P* = 0.016) in the two baseline groups. The SAPT group used fewer Ankura™II (*P* = 0.015) from Life tech. No significant differences were found in other baseline characteristics. As summarized in Table 2.

**Table 2:** Baseline characteristics, history, and laboratory tests between the two groups

Variable	Total (*n* = 245)	Group		*P*
		SAPT (*n* = 159)	DAPT (*n* = 86)	
Age, (year)	55.2 ± 12.9	55.2 ± 13.5	55.2 ± 12.0	0.999
Female, n (%)	37 (15.1)	25 (15.7)	12 (13.9)	0.712
Drinker, n (%)	58 (24.1)	35 (22.2)	23 (27.4)	0.379
Smoker, n (%)	88 (36.5)	56 (35.7)	32 (38.1)	0.709
Blood Transfusion Record, n (%)	8 (3.3)	5 (3.1)	3 (3.5)	1.000
Surgical History, n (%)	90 (36.9)	64 (40.5)	26 (30.2)	0.112
Previous Chronic Conditions, n (%)	16 (6.6)	11 (6.9)	5 (5.9)	0.755
Diabetes, n (%)	14 (5.7)	8 (5.0)	6 (6.9)	0.736
Hypertension, n (%)	185 (75.5)	118 (74.2)	67 (77.9)	0.521
Fenestrated TEVAR, n (%)	86(35.1)	46(28.9)	40(46.5)	0.006*
Single branched stent graft, n (%)	124(50.6)	84(52.8)	40(46.5)	0.345
Carotid subclavian bypass, n (%)	35(14.2)	29(18.2)	6(7.0)	0.016*
Lifetech Ankura™II	89 (36.3)	49 (30.8)	40 (46.5)	0.015*
GORE^®^	21 (8.6)	15 (9.4)	6 (6.9)	0.512
Mico Port Castor^®^	132(53.9)	92 (57.9)	40 (46.5)	0.089
COOK^®^	3 (1.2)	3 (1.9)	0 (0.0)	0.501
Diastolic Pressure, (mmHg)	82.3 ± 15.9	82.7 ± 15.2	81.5 ± 17.1	0.570
Systolic Pressure, (mmHg)	143.9 ± 25.5	144.2 ± 24.8	143.3 ± 27.0	0.807
BMI, (kg/m2)	25.5 (23.5–27.7)	25.3 (23.5–27.6)	25.7 (23.6–27.7)	0.570
Hospital Days, (day)	16.0 (12.0–21.0)	17.0 (12.0–22.0)	16.0 (13.0–19.0)	0.561
Operation Time, (min)	170.0 (120.0–290.0)	160.0 (120.0–292.5)	180.0 (131.2–275.0)	0.246
Platelet Count, (10^9/L)	188.1 ± 66.7	187.2 ± 67.9	189.8 ± 64.8	0.779
Hemoglobin Count, (g/L)	135.0 (122.0–146.0)	134.0 (121.0–145.0)	137.0 (123.0–146.5)	0.363
PT, (s)	11.4 (10.9–12.1)	11.5 (11.0–12.1)	11.4 (10.9–12.0)	0.547
INR, (s)	1.00 (0.9–1.1)	1.00 (0.9–1.1)	1.00 (0.9–1.1)	0.698
APTT, (s)	27.1 (25.6–29.1)	27.4 (25.9–29.3)	26.8 (25.1–28.8)	0.100
TT, (s)	17.3 (16.5–18.2)	17.4 (16.7–18.3)	17.2 (16.3–18.0)	0.106
FBG, (g/L)	2.8 (2.3–4.0)	2.8 (2.3–4.0)	2.8 (2.4–4.1)	0.782
D-D, (mg/L)	3.3 (1.6–6.9)	3.3 (1.16–7.80)	3.2 (1.9–6.5)	0.761

BMI: Body Mass Index PT: Prothrombin time; INR: International normalized ratio; APTT: Activated partial thromboplastin time; TT: Thrombin time; FBG: Fibrinogen; D-D: D-dimer; min: minute; s: second. * Statistically significant values

### Clinical outcome

During the follow-up, one patient was lost to follow-up, and 11 patients died. Among them, 8 patients (5.1%) received aspirin treatment, while 3 patients (3.5%) received DAPT with aspirin and clopidogrel (*P* = 0.808). Further details are summarized in Table 3. A total of 39 patients experienced hemorrhagic events, with 13 patients (9 BARC 1 type and 4 BARC 2 type) in the SAPT group, and 26 patients (13 BARC 1 type, 12 BARC 2 type, and 1 BARC 3 type) in the DAPT group, showing significant statistical difference (*P* < 0.001). There were no significant differences observed in other endpoints.

**Table 3:** Clinical outcomes follow-up between the two groups

Variable	Total (*n* = 245)	Group		*P*
		SAPT (*n* = 159)	DAPT (*n* = 86)	
Ischemic event, n (%)	59 (24.2)	38 (24.1)	21 (24.4)	0.949
Ischemic stroke, n (%)	3 (1.2)	3 (1.9)	0 (0.0)	0.498
Left arm ischemia, n (%)	17 (6.9)	10 (6.3)	7 (8.1)	0.596
Thromboembolic events, n (%)	41 (17.5)	27 (17.9)	14 (16.7)	0.814
Bleeding event, n (%)	39 (15.9)	13 (8.2)	26 (30.2)	<0.001*
BARC type1, n (%)	22 (9.1)	9 (5.7)	13 (15.5)	0.014*
BARC type2, n (%)	16 (6.6)	4 (2.5)	12 (14.3)	0.001*
BARC type3, n (%)	1 (0.4)	0 (0.0)	1 (1.2)	0.352
Death, n (%)	11 (4.5)	8 (5.1)	3 (3.5)	0.808
Cardiogenic death, n (%)	4 (1.6)	2 (1.3)	2 (2.3)	1.000
Aortic death, n (%)	5 (2.1)	1 (0.6)	1 (1.2)	1.000
Other causes of death, n (%)	2 (0.8)	5 (3.2)	0 (0.0)	1.000
Endoleak, n (%)	4 (1.7)	4 (2.6)	0 (0.0)	0.343

BARC: Bleeding Academic Research Consortium. *Statistically significant values

### Survival analysis of hemorrhagic and ischemic events

**Fig. 2:**
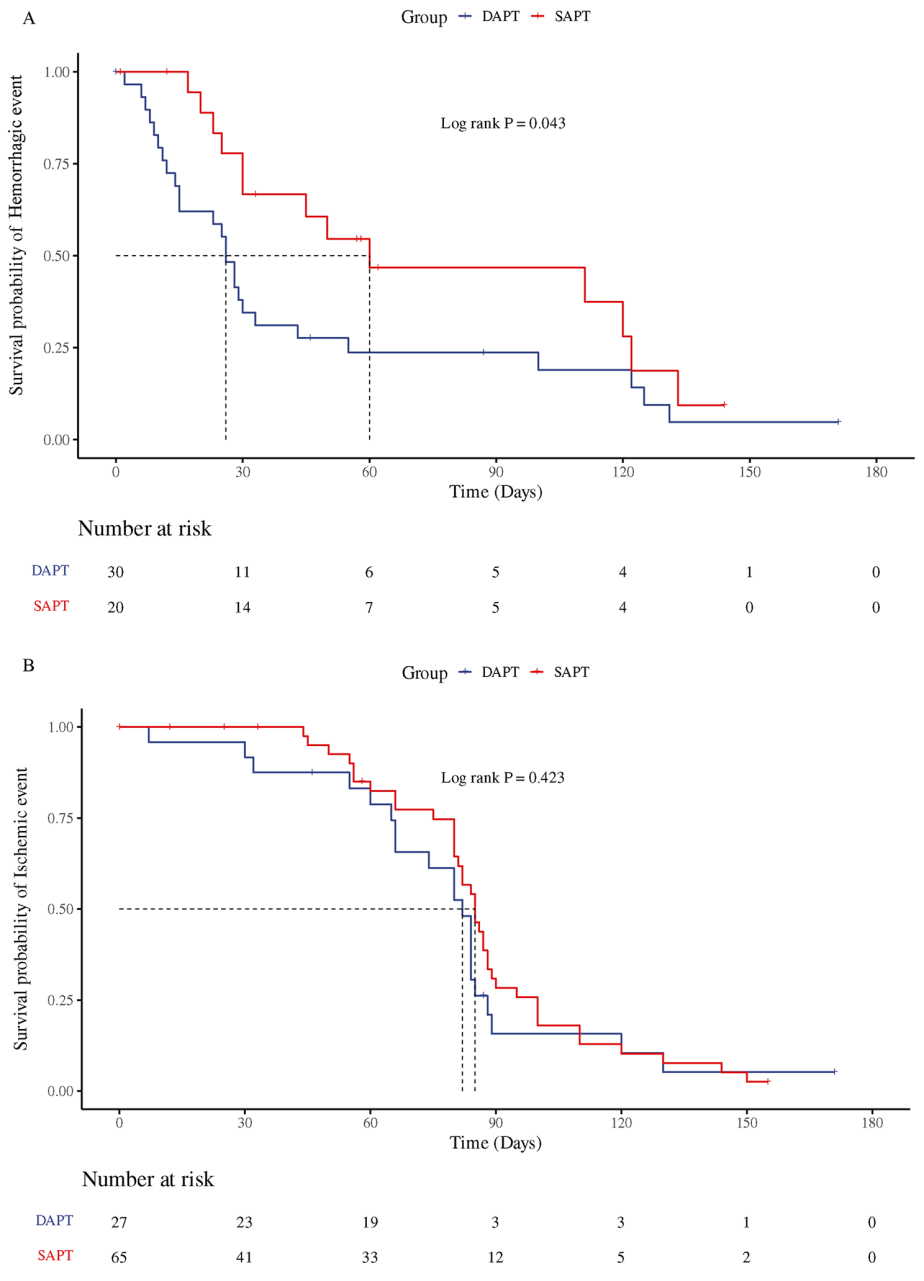
Survival analysis of hemorrhagic and ischemic events. DAPT = dual-antiplatelet therapy. SAPT = single-antiplatelet therapy. (**A**) Analysis results of hemorrhagic event. (**B**) Analysis results of ischemic event

Survival analysis revealed a lower risk of bleeding associated with SAPT during hemorrhagic events (*P* = 0.043). There was no significant difference in ischemic events between different antiplatelet regimens. Refer to Fig. 2 for a visualization of the data.

### Cox regression analysis of influencing factors of bleeding and ischemic events

**Fig. 3:**
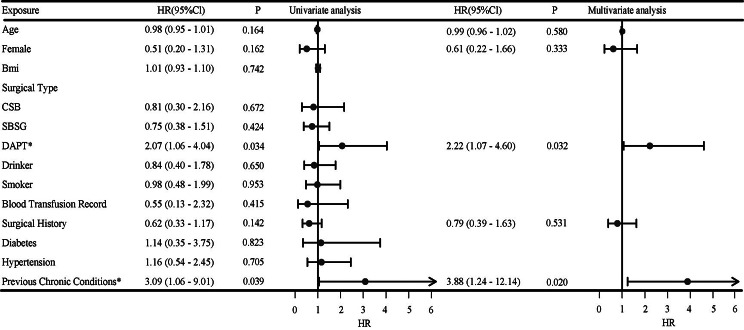
Forest map of univariate and multivariate hemorrhagic events

Utilizing binary Cox regression analysis to examine the influencing factors of hemorrhagic and ischemic events. Patient age, female, BMI, surgical type, DAPT, drinker, smoker, blood transfusion record, surgical history, diabetes, hypertension, and previous chronic conditions were included in the univariate regression model, with variables showing a significance of *P* < 0.2 included in the multivariate analysis. In the univariate analysis of bleeding events, variables such as age, female, DAPT, surgical history, and previous chronic conditions are the criteria for inclusion in the multivariable analysis. Among them, DAPT (HR: 2.07, 95% CI: 1.06–4.04, *P* = 0.034) and previous chronic conditions (HR: 3.09, 95% CI: 1.06–9.01, *P* = 0.039) showed a significant difference. The results of a multivariate analysis indicate a significant increase in the risk of bleeding with DAPT (HR: 2.22, 95% CI: 1.07–4.60, *P* = 0.032) and previous chronic conditions (HR: 3.88, 95% CI: 1.24–12.14, *P* = 0.020). Further details are summarized in Fig. 3.

**Fig. 4:**
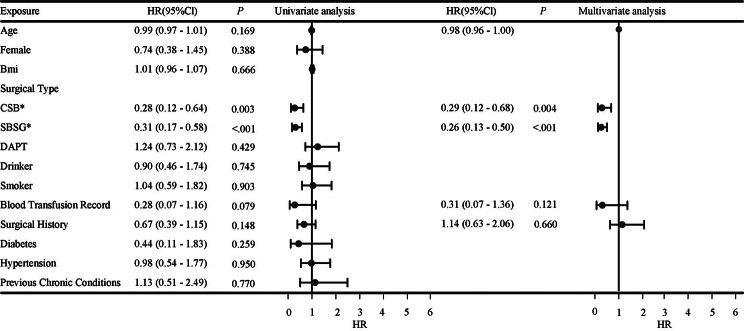
Forest map of univariate and multivariate ischemic events

In the univariate analysis of ischemic events, variables such as age, CSB, SBSG, blood transfusion record and surgical history are the criteria for inclusion in the multivariable analysis. Among them, CSB (HR: 0.28, 95% CI: 0,12 − 0,64, *P* = 0.003) and SBSG (HR: 0.31, 95% CI:0.17–0.58, *P* < 0.001) showed a significant difference. The results of a multivariate analysis indicate a significant reduction in the risk of ischemic events with CSB (HR: 0.29, 95% CI: 0,12 − 0,68, *P* = 0.004) and SBSG (HR: 0.26, 95% CI: 0,13 − 0,50, *P* < 0.001) compared to F-TEVAR. Further details are summarized in Fig. 4.

## Discussion

In type B aortic dissections, endovascular repair is commonly employed, for B dissections involving only the descending aorta, traditional straight tube stents are often used, with nearly 40% of cases involving the left subclavian artery. Reconstructing the left subclavian artery during TEVAR has gained wide recognition. Huang et al. [20], highlighted in a study evaluating the role of left subclavian artery revascularization in TEVAR that revascularization reduces the incidence of perioperative stroke and spinal cord ischemia (SCI), recommending its use in patients with LSA coverage during TEVAR. Leonhard-Bradshaw et al. [21], in their research assessing whether to reconstruct the left subclavian artery during TEVAR, they concluded that covering the LSA without revascularization increases the risk of stroke and SCI. Kimberly-Zamor et al. [22], proposed in a study comparing the incidence of LSA coverage during TEVAR and revascularization techniques that LSA coverage without revascularization during TEVAR increases the risk of stroke and upper limb ischemia. Methods of reconstructing the LSA artery during TEVAR include carotid subclavian bypass, fenestration stents, and castor-integrated single-branch stents [23]. However, due to narrow stent diameters and small artificial vessel calibers, stenosis and occlusion leading to ischemic events frequently occur.

The clinical application of antiplatelet therapy following LSA revascularization during TEVAR to prevent thromboembolic events originates from extensive research and understanding of antiplatelet inhibition in acute coronary syndrome and post-percutaneous coronary intervention (PCI) platelet activation [24]. Nevertheless, there are differences between TEVAR and PCI procedures. While antiplatelet therapy is generally administered post-LSA reconstruction, there is limited research on the use of antiplatelet therapy in patients undergoing left subclavian artery revascularization, with postoperative antiplatelet treatment strategies largely reliant on clinical judgment, lacking a consensus on their feasibility and safety.

To reduce the occurrence of ischemic events, antiplatelet therapy is often initiated postoperatively, albeit at the cost of increasing bleeding risk. Rational medication following LSA reconstruction can effectively improve patient prognosis. The occurrence of ischemic events such as stroke is one of the main risks after TEVAR surgery. Previous literature reports a lower incidence of stroke in patients undergoing LSA revascularization compared to those without LSA flow restoration [25]. However, there is currently a lack of research on the relationship between postoperative stroke occurrence in patients undergoing LSA reconstruction surgery and different antiplatelet treatment strategies. We found in this regression study that the risk of ischemic events was lower in CSB and SBSG reconstructive LSA approaches than in F-TEVAR, which is consistent with the results of a single-center study [23]. The use of DAPT was found to be ineffective in reducing ischemic events and increased the risk of bleeding within the 6-month study.

The majority of bleeding events in our study were non-disabling or non-fatal upper gastrointestinal bleeding, with fewer cases of severe bleeding such as hemorrhagic stroke, classified according to the US BARC classification. The results of the multivariate regression analysis showed that bleeding events in patients were not associated with increasing age, with dual antiplatelet therapy being the primary influencing factor to increase the risk of bleeding. Survival analysis results indicate that patients experience bleeding events mostly within 2 months, with an elevated risk of platelet bleeding associated with DAPT. Combining previous literature on post-percutaneous coronary intervention (PCI) [26] and post-peripheral arterial disease (PAD) studies [27], DAPT has been found to increase the risk of bleeding. The ideal clinical outcome is to reduce bleeding risk while preventing ischemic adverse events. In our study, the results were better with aspirin monotherapy, but in recent years, there have been more choices for monotherapy antiplatelet drugs, with clopidogrel monotherapy gradually replacing aspirin. In a study on post-PCI antiplatelet therapy, clopidogrel monotherapy was associated with a lower risk of bleeding compared to aspirin. Clopidogrel also showed greater advantages in balancing thrombosis formation and bleeding risk, but further substantial data is required for confirmation [28]. Our multivariate regression analysis results showed that a history of chronic diseases in patients increased the risk of ischemic events, necessitating further research and validation.

Our study results indicate that routine use of DAPT after LSA reconstruction does not provide additional benefits but increases the risk of bleeding. Therefore, our study supports that postoperative SAPT is a better therapeutic approach for patients. Patients with chronic diseases have a higher risk of ischemia. Decisions regarding antiplatelet drug use postoperatively should be made based on individual circumstances, with dynamic risk assessment of patients, weighing the cardiovascular event benefits against bleeding risks.

### Limitation

The study has limitations. Due to the inclusion criteria, some patients with underlying diseases were excluded, and the results of the study could not be extended to these groups. The study is a single-center regression analysis. The study includes a relatively small number of patients, and the follow-up period is insufficient. Some patients may not adhere to the prescribed medication schedule and dosage. Subsequent research should consider enlarging the sample size to validate these findings.

## Conclusion

In type B aortic dissection patients undergoing LSA blood flow reconstruction after synchronous TEVAR, the bleeding risk significantly decreases with the SAPT regimen, and there is no apparent ischemic compensation within 6 months. Patients with previous chronic conditions have a higher risk of bleeding. The CSB group and SBSG group have less ischemic risk compared to F-TEVAR group.

## Data Availability

The raw data supporting the conclusions of this article will be made available by the authors without undue reservation.

## Abbreviations

TEVAR: Thoracic endovascular aortic repair
LSA: Left subclavian artery
SAPT: Single antiplatelet therapy
DAPT: Dual antiplatelet therapy
DTAA: Descending thoracic aortic aneurysms
SCT: Subclavian-carotid transposition
CSB: Carotid subclavian bypass
TIA: Transient ischemic attack
EVAR: Endovascular abdominal aortic repair
F-TEVAR: Fenestration TEVAR
SBSG: Single-branched stent graft
BARC: Bleeding Academic Research Consortium
SCI: Spinal cord ischemia
PCI: Percutaneous coronary intervention
PAD: Post peripheral arterial disease

## References

[1] Volodos NL, Karpovich IP, Troyan VI, Kalashnikova Y, Shekhanin VE, Ternyuk NE, Neoneta AS, Ustinov NI, Yakovenko LF (1991). Clinical experience of the use of self-fixing synthetic prostheses for remote endoprosthetics of the thoracic and the abdominal aorta and iliac arteries through the femoral artery and as intraoperative endoprosthesis for Aorta Reconstruction. Vasa Suppl.

[2] Xie W, Xue Y, Li S, Jin M, Zhou Q, Wang D (2021). Left subclavian artery revascularization in thoracic endovascular aortic repair: single Center’s clinical experiences from 171 patients. J CARDIOTHORAC SURG.

[3] Hiratzka LF, Bakris GL, Beckman JA, Bersin RM, Carr VF, Casey DJ, A Report of the American College of Cardiology Foundation/American Heart Association Task Force On Practice Guidelines, American Association for Thoracic Surgery. 2010 Accf/Aha/Aats/Acr/Asa/Sca/Scai/Sir/Sts/Svm Guidelines for the Diagnosis and Management of Patients with Thoracic Aortic Disease., American College of Radiology,American Stroke Association, Society of Cardiovascular Anesthesiologists, Society for Cardiovascular Angiography and Interventions, Society of Interventional Radiology, Society of Thoracic Surgeons,and Society for Vascular Medicine. J AM Coll Cardiol. 2010; 55(14):e27-e129. 10.1016/j.jacc.2010.02.015. 10.1016/j.jacc.2010.02.015 20359588

[4] Riambau V, Bockler D, Brunkwall J, Cao P, Chiesa R, Coppi G, et al. editors. ‘s Choice - Management of Descending Thoracic Aorta Diseases: Clinical Practice Guidelines of the European Society for Vascular Surgery (Esvs). Eur J Vasc Endovasc. 2017; 53(1):4–52. 10.1016/j.ejvs.2016.06.005. 10.1016/j.ejvs.2016.06.005 28081802

[5] Upchurch GJ, Escobar GA, Azizzadeh A, Beck AW, Conrad MF, Matsumura JS (2021). Society for vascular surgery clinical practice guidelines of thoracic endovascular aortic repair for descending thoracic aortic aneurysms. J VASC SURG.

[6] Luehr M, Etz CD, Berezowski M, Nozdrzykowski M, Jerkku T, Peterss S (2019). Outcomes after thoracic endovascular aortic repair with overstenting of the left subclavian artery. ANN THORAC SURG.

[7] Matsumura JS, Lee WA, Mitchell RS, Farber MA, Murad MH, Lumsden AB, Greenberg RK, Safi HJ, Fairman RM (2009). The Society for vascular surgery practice guidelines: management of the left subclavian artery with thoracic endovascular aortic repair. J VASC SURG.

[8] van der Vliet JA, Palamba HW, Scharn DM, van Roye SF, Buskens FG (1995). Arterial Reconstruction for Subclavian Obstructive Disease: a comparison of extrathoracic procedures. EUR J VASC ENDOVASC.

[9] Canaud L, Morishita K, Gandet T, Sfeir J, Bommart S, Alric P, Mandelli M (2018). Homemade Fenestrated stent-graft for thoracic endovascular aortic repair of Zone 2 aortic lesions. J THORAC CARDIOV SUR.

[10] Jing Z, Lu Q, Feng J, Zhou J, Feng R, Zhao Z (2020). Endovascular repair of aortic dissection involving the left subclavian artery by Castor Stent Graft: a Multicentre prospective trial. EUR J VASC ENDOVASC.

[11] Boakye E, Uddin S, Obisesan OH, Osei AD, Dzaye O, Sharma G, McEvoy JW, Blumenthal R, Blaha MJ (2021). Aspirin for Cardiovascular Disease Prevention among adults in the United States: Trends, Prevalence, and participant characteristics Associated with Use. Am J Prev Cardiol.

[12] Rhee TG, Kumar M, Ross JS, Coll PP (2021). Age-related trajectories of Cardiovascular Risk and Use of Aspirin and Statin among U.S. adults aged 50 or older, 2011–2018. J AM GERIATR SOC.

[13] Zheng SL, Roddick AJ (2019). Association of Aspirin Use for Primary Prevention with Cardiovascular events and bleeding events: a systematic review and Meta-analysis. JAMA-J AM MED ASSOC.

[14] Calderone D, Greco A, Ingala S, Agnello F, Franchina G, Scalia L, Buccheri S, Capodanno D (2022). Efficacy and safety of aspirin for Primary Cardiovascular Risk Prevention in younger and older age: an updated systematic review and Meta-analysis of 173,810 subjects from 21 Randomized studies. THROMB HAEMOSTASIS.

[15] Pan Y, Elm JJ, Li H, Easton JD, Wang Y, Farrant M (2019). Outcomes Associated with Clopidogrel-Aspirin Use in Minor Stroke or transient ischemic attack: a pooled analysis of Clopidogrel in High-Risk patients with Acute Non-disabling cerebrovascular events (chance) and platelet-oriented inhibition in New Tia and minor ischemic stroke (point) trials. JAMA NEUROL.

[16] Johnston SC, Easton JD, Farrant M, Barsan W, Conwit RA, Elm JJ, Kim AS, Lindblad AS, Palesch YY (2018). Clopidogrel and Aspirin in Acute Ischemic Stroke and High-Risk Tia. NEW ENGL J MED.

[17] He RX, Zhang L, Zhou TN, Yuan WJ, Liu YJ, Fu WX, Jing QM, Liu HW, Wang XZ (2017). Safety and Necessity of Antiplatelet Therapy on patients underwent endovascular aortic repair with both Stanford Type B aortic dissection and Coronary Heart Disease. Chin MED J-PEKING.

[18] Hansson EC, Geirsson A, Hjortdal V, Mennander A, Olsson C, Gunn J (2019). Preoperative dual antiplatelet therapy increases bleeding and transfusions but not mortality in Acute Aortic dissection type a repair. EUR J CARDIO-THORAC.

[19] Mehran R, Rao SV, Bhatt DL, Gibson CM, Caixeta A, Eikelboom J (2011). Standardized bleeding definitions for Cardiovascular clinical trials: a Consensus Report from the Bleeding Academic Research Consortium. Circulation.

[20] Huang Q, Chen XM, Yang H, Lin QN, Qin X (2018). Effect of left subclavian artery revascularisation in thoracic endovascular aortic repair: a systematic review and Meta-analysis. EUR J VASC ENDOVASC.

[21] Bradshaw RJ, Ahanchi SS, Powell O, Larion S, Brandt C, Soult MC, Panneton JM (2017). Left subclavian artery revascularization in Zone 2 thoracic endovascular aortic repair is Associated with Lower Stroke Risk Across All Aortic diseases. J VASC SURG.

[22] Zamor KC, Eskandari MK, Rodriguez HE, Ho KJ, Morasch MD, Hoel AW (2015). Outcomes of thoracic endovascular aortic repair and subclavian revascularization techniques. J AM COLL Surg.

[23] Wu X, Li Y, Zhao Y, Zhu Y, Wang S, Ma Q (2023). Efficacy of left subclavian artery revascularization strategies during thoracic endovascular aortic repair in patients with type B dissection: a single-center experience of 105 patients. FRONT CARDIOVASC MED.

[24] Capodanno D, Alfonso F, Levine GN, Valgimigli M, Angiolillo DJ. Acc/Aha Versus Esc Guidelines On Dual Antiplatelet Therapy: Jacc Guideline Comparison. J Am Coll Cardiol. 2018; 72(23 Pt A):2915–31. 10.1016/j.jacc.2018.09.057. 10.1016/j.jacc.2018.09.057 30522654

[25] von Allmen RS, Gahl B, Powell JT, editors. ‘s Choice - Incidence of Stroke Following Thoracic Endovascular Aortic Repair for Descending Aortic Aneurysm: A Systematic Review of the Literature with Meta-Analysis. Eur J Vasc Endovasc. 2017; 53(2):176–84. 10.1016/j.ejvs.2016.10.025. 10.1016/j.ejvs.2016.10.025 27993454

[26] Capranzano P, Moliterno D, Capodanno D (2024). Aspirin-free antiplatelet strategies after percutaneous coronary interventions. EUR HEART J.

[27] Beiswenger AC, Jo A, Harth K, Kumins NH, Shishehbor MH, Kashyap VS (2018). A systematic review of the efficacy of aspirin Monotherapy Versus Other Antiplatelet Therapy regimens in Peripheral arterial disease. J VASC SURG.

[28] Yang S, Kang J, Park KW, Hur SH, Lee NH, Hwang D (2023). Comparison of Antiplatelet monotherapies after percutaneous coronary intervention according to clinical, ischemic, and bleeding risks. J AM COLL CARDIOL.

